# The Adoption and Acceptance of mHealth Interventions for Self-Management of Hypertension Among Adult Patients: A Systematic Review

**DOI:** 10.7759/cureus.31584

**Published:** 2022-11-16

**Authors:** Samer A Alzahrani, Mohammed F Bin Muammar, Abdullah F Bin Muammar, Ahmed Alolah, Mohammed Almutawa

**Affiliations:** 1 Medicine, Imam Mohammad Ibn Saud Islamic University (IMSIU), Riyadh, SAU; 2 Medicine and Surgery, Imam Mohammad Ibn Saud Islamic University (IMSIU), Riyadh, SAU; 3 Medicine and Surgery, King Saud University, Riyadh, SAU; 4 Medicine and Surgery, Prince Sattam bin Abdulaziz University, Riyadh, SAU

**Keywords:** systematic review, implementing health innovation, medicine and mobile technology, self-care, hypertension

## Abstract

Hypertension is a worldwide epidemic that affects healthcare costs and public health. As a result, self-management of this disease and, in this context, mobile health (mHealth) can be used as a cost-effective management tool. Self-management of hypertension remains of great significance due to the rising number of hypertension cases. As a result, this study aimed to assess the various mobile health interventions used in the self-management of hypertension, their user acceptability, compliance, and adherence to hypertension treatment, and their effectiveness. Some mobile health techniques are automated text and video messages. These mobile applications allow for self-monitoring and communication between the patients and the health service providers, reminders, and automated signals. The abovementioned interventions are promising tools in helping manage blood pressure (BP), but resources are limited. This review involved selecting studies associated with mobile health interventions in managing hypertension and extracting data from available resources. Thirteen studies were selected using the inclusion criteria, and relevant data were extracted and discussed in the review. This review reported the role of mobile health interventions in the management of blood pressure, as most studies noted a decrease in blood pressure and increased medication adherence and self-efficacy. It also reported a reliable communication channel between the participants and their health service providers.

## Introduction and background

Hypertension is prevalent among the human population across the world [1]. Hypertension is a condition in which pressure in the blood vessels is persistently raised. It happens when blood moves through the arteries at a higher pressure than normal. The European Society of Cardiology has defined hypertension as systolic blood pressure (SBP) ≥ 140 mm Hg and/or diastolic blood pressure (DBP) ≥ 90 mm Hg [2]. Hypertension has been known as a significant risk factor for cardiovascular diseases since the 1960s [3]. Hypertension can be classified as either primary or secondary hypertension. Primary hypertension is defined as blood pressure (BP) ≥ 140/90 mm Hg in the absence of an identifiable secondary cause [4]. It is usually diagnosed by a doctor after three or more episodes of high blood pressure, having eliminated all the other causes of blood pressure. Although the etiology is disputable, researchers suggest that old age, heredity, smoking, diet, and obesity are primarily associated with the occurrence of hypertension [5].

On the other hand, secondary hypertension is identified as high blood pressure caused by underlying medical conditions, including cardiovascular issues, kidney problems, thyroid disease, tumors of the adrenal glands, alcohol, and excessive salt intake. In addition, some drugs, such as ibuprofen and pseudoephedrine, are available over the counter and have been associated with secondary hypertension [6]. Additional hypertension types with specific diagnostic criteria have also been documented by Chris Iliades, including detached systolic hypertension, malignant hypertension, and resistant hypertension [6]. Individuals exceeding the age of 65 years are at risk of isolated systolic hypertension because the elasticity of the arteries is lost. Thus, the blood pressure is usually recorded using two numerals, the upper numerals (systolic pressure) and the lower numerals (diastolic pressure). With isolated systolic pressure, the systolic pressure has to rise beyond 140 mm Hg, but diastolic pressure is almost at the optimal 90 mm Hg.

Malignant hypertension, on the other hand, occurs in the young population and females with pregnancy toxemia. It is experienced mainly when systolic blood pressure is elevated to above 180 mm Hg and diastolic blood pressure above 120 mm Hg [7]. The symptoms of malignant hypertension are related to end-organ damage, including headaches, nausea or vomiting, visual disturbances, chest or back pain, dyspnea, orthopnea, or visual disturbances [8]. Resistant hypertension occurs if the blood pressure is still high despite taking three antihypertensive drugs [9]. It occurs mostly in the elderly who might have some genetic component with underlying kidney disease or diabetes [10]. Additionally, hypertension can cause numerous clinically devastating complications, such as ischemic heart disease, heart failure, and aortic aneurysm, which require early diagnosis and treatment to be avoided [8]. The management of hypertension mainly involves self-care in addition to diuretics. Eating healthy with diets that consist of small amounts of sodium, exercising, managing stress, and taking medications such as diuretics or vasodilators can help lower blood pressure [11,12]. Self-care and management, however, is the most used method in managing hypertension alongside medication [13]. As a result, it has led to the development and use of information and internet-based applications and interventions to manage hypertension.

Patients and caregivers have used electronic and communication technologies in the self-management of chronic illnesses. Mobile health technology, frequently referred to as mHealth, involves using tablets, smartphones, and personal computers to manage chronic diseases such as hypertension. There is increased availability of smartphone applications and website platforms that help people monitor and manage their hypertension [14]. Such interventions are referred to as eHealth and are based on mobile health and telemedicine. Mobile health has the potential to complement the doctor’s intervention and promote patients’ self-management [15]. An example of the application of telemedicine for the management of hypertension is telemonitoring, which allows for the remote data transmission of blood pressure and patient information from their living site to the hospital or the doctor’s office [16]. A significant reduction in blood pressure among high-risk hypertensive patients after the use of mobile health techniques compared to usual care was further noted. Additional benefits have been described when blood pressure telemonitoring is offered under the supervision of healthcare personnel. Optimization of drug use, maintenance of a log of blood pressure measurements, improved compliance to treatment, improved quality of life, and reduced risk of cardiovascular complications have been discussed as benefits of using mobile health technology in managing hypertension. The effective treatment of hypertension requires self-management, and with the development of mobile technologies, mHealth has become so crucial in the management of hypertension [16]. The effectiveness of mobile health in managing hypertension among patients was also reported, noting improved medication adherence and self-management behavior. Li et al. reported the most successful mHealth intervention that combined tailored messages, interactive communication, and multifaceted functions [16]. Indraratna et al. noted a significantly lower systolic blood pressure among patients with hypertension exposed to mobile phone intervention. Furthermore, they demonstrated its effectiveness in managing hypertension among other conditions such as heart failure [17]. mHealth systems help patients manage their hypertension in different ways, including reminders and alarms to take their medication, linking their blood pressure to their doctors for review, and providing blood pressure feedback reports to the patients [18]. This review is designed to appraise the adoption and acceptance of mHealth technology interventions, user satisfaction, and compliance in the self-management of hypertension.

## Review

Methodology

Design

This systematic analysis was conducted in line with the Cochrane approach with a report given using approved presentation tools used in Preferred Reporting Items for Systematic Reviews and Meta-Analyses (PRISMA).

Literature Search

We searched various databases for studies using electronic methods. The main databases were PubMed (October 21, 2022), Cochrane Central Register of Controlled Trials (CENTRAL) (October 22, 2022), and Excerpta Medica database (EMBASE) (October 22, 2022). Any study published from the inception of this systematic review (October 2022) was included as part of the lists of retrieved studies submitted and checked for inclusion eligibility after that. A detailed electronic search was performed using keywords and Medical Subject Headings (MeSH) terms. We also used Boolean operators, including truncation (asterisk), and field tags (tab and two) that generated variation for some search strings for studying identifications of databases. We used some of the following keywords that were combined, thereby forming the conceptualized search string used in the herein research study: (“mobile-health” albeit “mhealth technology” alternatively, “telehealth” together with “self-management” albeit “self-management” and “management” also, “hypertension” albeit, “high blood pressure”). We also incorporated the following MeSH terms in the search “mobile health” (MeSH) AND “management” (MeSH) AND “hypertension” (MeSH). We applied the search string to the bibliographic databases to identify good literature.

Eligibility Criteria

Two independent reviewers assessed the identified studies using predesigned items for eligibility.

Inclusion criteria: Studies were selected for inclusion if they were randomized controlled trials assessing the use of mHealth interventions in the management of hypertension and other chronic illnesses, studies assessing subjects with diagnosed hypertension, studies among hypertension patients using mHealth interventions for self-management, and studies that reported outcomes such as user satisfaction, user compliance, and the efficacy of mobile health interventions. There was no time limit in terms of the year of publication.

Exclusion criteria: The following studies were excluded: studies reporting other interventions for hypertension apart from mobile health interventions, those reporting the use of mobile health interventions in the management of other disease conditions apart from hypertension, and studies published in a non-English language that did not have an English translation.

Data Extraction

Data were extracted into a standardized Excel sheet (Microsoft Corp., Redmond, WA, USA) by two reviewers working independently. The data extracted included the first author (year), study design, mobile therapy applied for self-management, and outcomes in terms of efficacy, user compliance, and user satisfaction. Data regarding comparative improvements between mHealth interventions and standard care or standard education were also collected. In addition, data were revised for any differences, which were ironed out through deliberations with a third party.

Result Analysis

This systematic review adopted a single form of analysis for this investigation. We used qualitative assessment to expound on the findings presented by the included studies. Literature analysis was used while conducting a systematic review of evidence provided by the included studies. To analyze this literature, descriptive methods of analysis were adopted. The process involved constructively summarizing key data points to identify patterns within the findings. In that way, the review can show any conclusive conditions and even draw inferences from the information at hand.

Results

Study Selection

**Figure 1:**
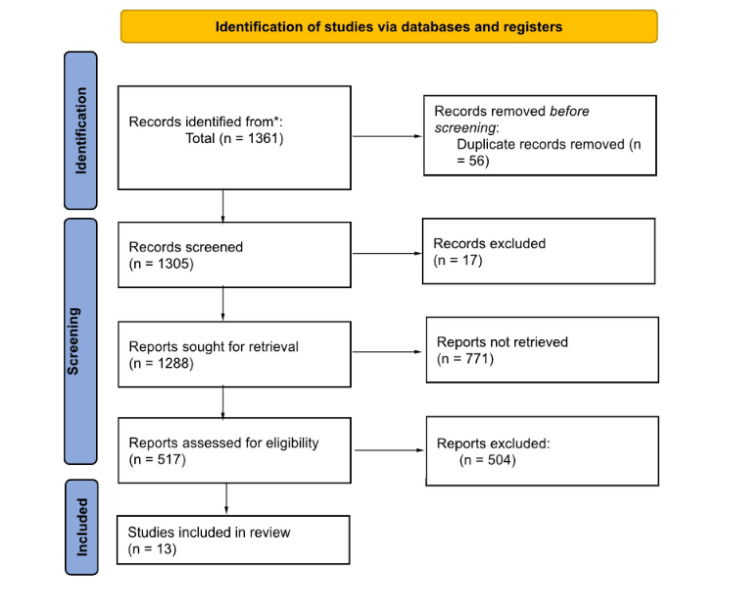
PRISMA flow diagram detailing the study selection process. PRISMA: Preferred Reporting Items for Systematic Reviews and Meta-Analyses

From the literature search, we found 1,361 studies published on self-management of hypertension after searching the databases and reference lists. Of all these, 56 studies were eliminated by automation tools for duplicity and other reasons for ineligibility. Only 1,305 studies were brought forth for the title and abstract screening, whereas 788 studies were eliminated, leaving 517 studies. Full-text screening of the 517 studies eliminated 504 studies for major methodological reasons such as reporting different outcomes, not being the eligible study design, reporting redundant findings, or containing incomplete or retracted results. Plot studies, study protocols, diagnoses, systematic reviews, and meta-analyses were also eliminated. We included 13 studies in this systematic literature review. Figure 1 presents a PRISMA 2020 flow diagram showing the study selection process.

All these 13 studies looked at different hypertensive adult patients under mobile health management interventions. Each of them studied the use of mHealth technology, which included communicating with the patients using text, video, or voice messages. A distinction between them was that some studies used mHealth to manage hypertension patients, while others used mHealth to monitor populations at a higher risk of hypertension, helping them avoid it. The bottom line is that all 13 included studies investigated the use of mHealth interventions in managing a hypertensive crisis (Table 1).

**Table 1:** Summary of the findings of the included studies. TASMINH4: Telemonitoring and Self-monitoring in Hypertension, SMS: short messaging service, BMI: body mass index, MA: medication adherence, BP: blood pressure, SBP: systolic blood pressure, DBP: diastolic blood pressure, SC: standard care, SD: standard deviation, BPMAP: Blood Pressure Management Application, CI: confidence interval

Author	Year	Study design	Aim	mHealth intervention	Outcome
Zha et al. [19]	2020	Randomized controlled trial	To assess the effectiveness of self-management and self-monitoring among hypertension patients in an urban community using a mobile health (mHealth) intervention	iHealth BP7 Wireless Blood Pressure Wrist Monitor (iHealth Lab Inc.)	The intervention group showed a significant improvement in systolic blood pressure reduction (p=0.01) with better adherence to medication, self-efficacy, and monitoring at six months, compared to the control group.
Grant et al. [20]	2019	Randomized controlled trial	To evaluate facilitators and barriers to self- and telemonitoring interventions for hypertension within the TASMINH4 trial	Free short text messages (SMS) that were employed for self-monitoring	Structured home monitoring engaged and empowered patients to self-monitor. Mobile health provided a safe, rapid, and reliable communication channel between the intervention group and their health service providers. It also provides additional benefits to practices over self-monitoring, although both need to be integrated for better results and to ensure generalizability.
Moon et al. [21]	2019	Randomized controlled trial	To compare the fidelity of a mHealth intervention using a smartphone application and a handwritten logbook to make hypertension patients’ data available to medical personnel for easier follow-ups	Smartphone application	The fidelity of the smartphone application was significantly higher in the first week (64.4%) than in the second (55.1%, p=0.001) and third (58.2%, p=0.03) weeks of monitoring. Although fidelity was higher among the application users, there was no significant difference between study arms (smartphone application: 66.7%; logbook: 52.4%; p=0.21).
Bernabe-Ortiz et al. [22]	2020	Randomized controlled trial	To assess the effects of mHealth intervention for one year on body weight and blood pressure levels in a low-resource population in Peru	Tailored SMS text messages, motivational interview calls, and messaging	There was a significant decrease in body weight and BMI after five years of randomization. However, there were no effects on blood pressure levels noted. Therefore, mHealth seems to be a potential preventive strategy for chronic and noncommunicable diseases in resource-constrained settings.
Chandler et al. [23]	2019	Randomized controlled trial	To conduct a nine-month smartphone-enabled efficacy trial targeting blood pressure and medication adherence among Hispanic adults with uncontrolled hypertension and poor MA	SMASH (a smartphone application using programmed text and video messages)	SBP averages were significantly lower in the SMASH versus the standard control groups (month 1: 125.3 versus 140.6; month 3: 120.4 versus 137.5, month 6: 121.2 versus 145.7; month 9: 121.8 versus 145.7; p<0.01). The mHealth interventions resulted in an increased self-medical regimen and proved to be an effective method for medication adherence.
Davidson et al. [24]	2015	Randomized controlled trial	To assess and extract the findings of using a mHealth strategy, SMASH, among African American and Hispanic adults with uncontrolled essential hypertension after six months	SMASH (tailored smartphone messaging (SMS) and reminder signals)	There were significant reductions in blood pressure, both SBP and DBP, for the intervention group versus the SC control group across all time points. For example, 70.6% of SMASH subjects versus 15.8% of the SC group reached BP control (<140/90 mm Hg) at month 1 (p<0.001).
Dorsch et al. [25]	2020	Randomized controlled trial	To evaluate the effect of using a mobile application intervention, LowSalt4Life, on reducing sodium intake in adults with hypertension	Mobile app intervention (LowSalt4Life)	There was a notable decrease in urinary sodium levels in the estimated 24 hours calculated from the application group compared to the no application group (-462 (SD: 1,220) mg versus 381 (SD: 1,460) mg, respectively; p=0.03).
Bhandari et al. [26]	2022	Randomized controlled trial	To assess the acceptability, compliance, and effectiveness of a mobile phone text messaging intervention (TEXT4BP) to improve hypertension control and medication adherence and enhance self-management among hypertension patients in Nepal	Mobile phone text messaging (TEXT4BP)	At three months, the intervention group had greater reductions in SBP and DBP versus usual care (-7.09/-5.86 (p≤0.003) versus -0.77/-1.35 (p≥0.28) mm Hg). The intervention arm showed an improvement in compliance with antihypertensive therapy (p<0.001), medication adherence (p<0.001), medication adherence self-efficacy (p=0.023), and knowledge of hypertension and its treatment (p=0.013). Participants expressed a high acceptability rate and desire to continue the TEXT4BP intervention.
Bozorgi et al. [27]	2021	Randomized controlled trial	To evaluate the effectiveness of the BPMAP in promoting adherence to medical treatments in patients with primary hypertension in Iran	A mobile application-based educational-supportive intervention, along with routine treatment	The treatment adherence score increased by an average of 5.9 (95% CI: 5.0-6.7) in the intervention group compared to the control group. The scores of “adherence to the low-fat and low-salt diet plans” were 1.7 (95% CI: 1.3-2.1) and 1.5 (95% CI: 1.2-1.9), respectively. Additionally, physical activity increased to 100 minutes (95% CI: 61.7-138.3) per week in the study group.
Oh et al. [28]	2022	Randomized controlled trial	To evaluate the effects of an integrative mHealth intervention strategy in managing hypertension, obesity, and blood glucose levels to report clinical outcomes	Self-measuring home devices for monitoring blood pressure and blood glucose in integration with two smartphone applications	Among the three outcomes under evaluation of food intake, exercise, and medicine intake in smartphone applications, adherence to medicine intake was a more useful, easier-to-use, and better-designed function than the others. The input rates of food intake and exercise to the smartphone application were very low (24.9% and 5.3%, respectively). On the contrary, the input rate of medicine intake was high (84%). Moreover, there was no significant difference in the input rate of taking medicine irrespective of whether the mHealth period was before or after the conventional treatment period (80.3% and 87.3%, respectively; p=0.06).
Jahan et al. [29]	2020	Randomized controlled trial	To create awareness and knowledge to promote lifestyle behavior changes among individuals with hypertension in a rural community of Bangladesh using mobile health technology (mHealth)	Tailored SMS text messaging	Adherence rates were significantly higher (9%) among the control group regarding salt intake (p=0.04) and physical activity behaviors (p<0.03), and little differences were observed in other behaviors. Face-to-face health education requires integrating both interactive text messages and home care providers for greater effectiveness of the intervention strategy.
Barsky et al. [30]	2019	Randomized controlled trial	To assess the blood pressure reduction difference in the self-management of hypertension after using mHealth intervention using text messages (SMS)	Tailored SMS text messages	Bluetooth technology enabled fast, reliable, and safe transmission of information from participants to their healthcare providers and vice versa. In remote communities, the DREAM-GLOBAL study helped local healthcare providers help their patients in the management of hypertension, and it allowed the participants to feel more connected to their health service providers. Additionally, the technical components of the study were implemented as planned, and patients felt supported in their self-management through the SMS text messaging and mobile health program.
Muldoon et al. [31]	2020	Clinical trial	To test the hypothesis of whether the MyBP application will improve hypertension self-efficacy, improve medication adherence, and lower BP in older adults	BP self-monitoring application with automated text messages and communication between patients and health service providers	Hypertension medication adherence and self-efficacy improved in the intervention group compared to the control group (estimate 0.556 (0.104, 1.008)). There was a reported decrease in blood pressure if home blood pressure was increased at baseline.

Discussion

The most commonly reported health interventions are mobile applications with automated text and video messages and self-monitoring equipment. Various researchers have discussed a couple of outcomes, more appearing to be a reduction in blood pressure, systolic and diastolic blood pressure, user acceptance and user compliance, medication adherence, and reduced hypertensive symptoms. Of the 13 studies, 10 [19,20,22-26,29-31] have reported a significant reduction in blood pressure following the use of automated messages, video messages, self-monitoring applications, and mobile applications. Davidson et al. reported using reminders and signals as mobile health interventions among individuals with essential hypertension [24]. This can be attributed to increased user compliance in medication adherence and self-monitoring [18]. Bhandari et al. also reported decreased systolic and diastolic blood pressure in the intervention group that was exposed to automated text messages and video messages. In addition, the intervention arm showed an improvement in compliance with antihypertensive therapy (p<0.001), medication adherence (p<0.001), medication adherence self-efficacy (p=0.023), and knowledge of hypertension and its treatment (p=0.013) [26]. Chandler et al. also noted that systolic blood pressure averages were significantly lower in the SMASH versus control groups (first month: 125.3 versus 140.6; third month: 120.4 versus 137.5, sixth month: 121.2 versus 145.7; ninth month: 121.8 versus 145.7; p<0.01) [23].

Participants expressed a high acceptability rate and a desire to continue the TEXT4BP intervention [26]. This study reported positive user acceptance and the user’s willingness to continue with the mobile health intervention for self-management. Jahan et al. also reported user willingness to incorporate the mHealth technique, SMS text messages, as a mobile health monitoring and management method [29]. Some of the users, however, suggested that they required both home healthcare provision and face-to-face health education for more relevance and to increase the effectiveness of the timely and interactive text messages.

Dorsch et al. conducted a study on using a mobile application (LowSalt4Life) to reduce the salt intake of hypertensive individuals. As a result, there was a significant reduction in the urinary sodium excreted in the group that used the application compared to the no application group (-462 (SD: 1,220) mg versus 381 (SD: 1,460) mg; p=0.03) [25].

A couple of studies also noted an increase in treatment and medication adherence. The treatment adherence score increased by an average of 5.9 (95% CI: 5.0-6.7) in the intervention group compared to the control group. The scores of “adherence to low-fat and low-salt diet plans” were 1.7 (95% CI: 1.3-2.1) and 1.5 (95% CI: 1.2-1.9), respectively [27]. Additionally, the intervention group increased physical activity to 100 minutes (95% CI: 61.7-138.3) per week. This demonstrates the efficacy of mHealth interventions in self-management as there is a notable decrease in blood pressure following the correct medication adherence. Muldoon et al. reported improved self-efficacy in the intervention group compared to the control group with an estimate of 0.556 (0.104, 1.008) [31]. Furthermore, adherence rates were significantly higher (9%) among the intervention group regarding salt intake (p=0.04) [29]. Zha et al. also reported that the mHealth group had better blood pressure monitoring and improved medication adherence compared to the control group at six months. Self-monitoring also ensured proper communication between patients and medical experts [19]. Structured home monitoring empowered patients to self-monitor, which is illustrated by Grant et al. who reported increased and efficient communication between patients and medical personnel. They further reported that telemonitoring gave additional benefits to practices over and above self-monitoring, although both need to be offered for generalizability [20].

Barsky et al. further reported that using automated text messages and Bluetooth technology enabled the transmission of reliable and secure information between participants and health service providers. The participants also felt supported in their self-management through the SMS text messaging and mobile health program and expressed their willingness to continue with the program. They also cited the availability of fast and easy consultation services from their health service providers [30]. Zha et al. conducted a study using the iHealth BP7 Wireless Blood Pressure Wrist Monitor (iHealth Lab Inc.), which resulted in significant improvement in SBP decrease (p=0.01). The intervention group had better and improved medication adherence compared to the standard follow-up group. Although most studies (10/13) cited a significant reduction in blood pressure, some of the studies noted no difference at all. For example, Bernabe-Ortiz et al. stated that although there were no visible effects on blood pressure levels, other reductions had been recorded, including reductions in body weight and BMI after five years of randomization [22].

## Conclusions

This study was conducted to check for user satisfaction and user compliance with the currently available mHealth interventions. A couple of mobile health interventions have been mentioned earlier, including mobile applications, self-monitoring applications, automated text messages, and voice messages. All these interventions are disseminated to various patient groups with many variations. While most studies have noted a positive contribution of these mHealth interventions, some of the studies illustrated no significant difference between the mHealth groups and the control groups. However, 10 out of the 13 included studies have demonstrated a reduction in blood pressure following the use of mobile health interventions with increased medication adherence and self-efficacy among participants.

Furthermore, increased medication adherence has been linked to easy, fast, and available communication between the patients and their medical personnel, as most of these mHealth interventions promote reliable communication between the two groups. Therefore, medical service providers can track the patient’s blood pressure measurements and progress and advise accordingly. Furthermore, in remote areas, mobile health programs helped local service providers connect with their patients and follow through on their self-management journey.

Although smartphone applications have contributed to managing hypertension, they need a more integrated option that couples mobile health interventions and face-to-face medical treatment and services. The current spike in telecommunication technological advancements and internet connectivity makes the application of these mHealth interventions much easier. This systematic review has studied a number of past literature to determine the rate or rather the degree of adoption and acceptance of these technologies, but still, the endeavor was limited. For one, there are very few studies discussing the outcome of the adoption and acceptance of mHealth technology interventions. The closest outcome focused on by this review to assess the primary outcome was the adherence to the interventions and the impact of the interventions on the prevalence of hypertension crises. To effectively manage a hypertension crisis, it is important to understand how the patients adopt these interventions. By so doing, proper strategic implementations can be put forward to not only encourage the population to adhere to medication and a holistic lifestyle but also adopt the mHealth measures. Therefore, this review recommends that more empirical studies be carried out with the primary objective of discovering the perception of hypertension patients and the population in general toward these interventions.
